# Suicide: What the General Public and the Individual Should Know

**DOI:** 10.21315/mjms2018.25.2.2

**Published:** 2018-04-27

**Authors:** Saxby Pridmore, Tammie T Money, William Pridmore

**Affiliations:** 1 Discipline of Psychiatry, University of Tasmania, Hobart, TAS, Australia; 2 Post-Graduate Student, Australian National University Medical School, Garran, ACT, Australia

**Keywords:** suicide, suicide prevention, mental disorder

## Abstract

**Background:**

The predominant, current western view is that all suicide is the result of mental disorder. This view is much too narrow and does not admit extensive information regarding the social, economic, and forensic factors (among many others) which may contribute to completed suicide. A consequence of this narrow view is that prevention strategies mainly focus on the detection and treatment of mental disorder. A preferred approach is to place greater emphasis on public health approaches to suicide prevention.

**Objective:**

To develop and suggest a body of information which may be useful in a public health approach to suicide.

**Conclusion:**

It is suggested that the following be available to the general public: i) suicide is a fact of life which should be minimised, ii) suicide has many different triggers, iii) most people who take their lives are able to make decisions, and iv) increased public discussion and understanding of suicide is desirable. Five pieces of information that may be useful to those contemplating suicide include: i) don’t murder the part of you that wants to live, ii) suicide actions may leave you alive but disabled, iii) suicide hurts other people, iv) suicidal impulses do pass if you hold on, and v) suicide is a waste.

## Introduction

For the last two centuries, western wisdom has taught that all (or almost all) suicide is the result of mental disorder. This simplistic view led particular “experts” to be well supplied with authority, celebrity, and research funds. It limited our grasp of the numerous complexities of suicide and focused the vast majority of suicide prevention activities on the medical model and medical treatments. Recently, however, there have been calls for a public health approach to this behaviour.

In 1998, Rosenman (1) found that suicide prevention projects focused on people at high risk were ineffective and could even be making matters worse. He suggested a public health approach, including restricting access to lethal means, and the need “to address the social ills which produce morbidity, whether or not they lead to suicide”. In 2002, De Leo (2) remarked that no progress was being made in preventing suicide. He wrote of the need to consider socioeconomic and cultural factors, a multidisciplinary approach, and for “strategies targeting the general population instead of high-risk groups”. In 2010, Caine (3) admired the efforts of US clinicians but observed that in spite of them the suicide rate had risen; he opined, “We must foster the development of public health and preventive psychiatry”. In 2016, Reidenberg and Berman (4) found that the suicide rate in the US had increased; they called for “changing the direction of suicide prevention” and recommended public health strategies.

Public health has been defined as “the organised response by society to protect and promote health, and to prevent illness, injury, and disability. The starting point for identifying public health issues, problems, and priorities, and for designing and implementing interventions, is the population as a whole, or population sub-groups” (5). Successful campaigns have included reducing the transmission of AIDS, smoking, and deaths from road trauma. Education is an important component.

Rather than attempting to identify people at high risk of suicide (a failed prevention method (6, 7)), the public health approach involves the whole community in education and activities, including those who are at low risk (which is the group that comprises the majority of completed suicides).

Road deaths were reduced by public health activities, such as i) improved built environment (roads), ii) improved motor vehicles (better braking, air bags), iii) enforced regulations (speed and blood alcohol limits, roadworthiness of vehicles), and iv) driver education. Smoking was reduced by public health activities, such as i) increasing the cost of cigarettes; ii) reducing the attractiveness of packaging; iii) repeated, graphic illustrations of the pathology caused by smoking (pictures on packets, in some countries); and iv) reducing the opportunities to smoke (outlawing smoking inside at public events, etc.). In both cases, governments made changes to the environment and led a public education campaign. Although not directly promoted by governments, irresponsible driving and smoking ceased to be socially condoned and eventually became less common.

Evidence indicates that individuals who are contemplating suicide are experiencing emotional pain (8). They are located in distressing predicaments (9); these include mental illnesses, but also a wide range of social (10), economic (11), political (12), forensic (13), and intoxication (14) circumstances, from which other escape options are limited or not apparent to the individual.

## Aim

To offer some points which might be used in a public health approach to suicide.

## Method

A comprehensive review of the scientific suicide and public health literature. This was considered from the perspective of scholars with extensive experience in suicidology and public health.

## Results

We present our opinion points below under two headings: i) information for the general community and ii) information for the person with suicidal thoughts.

Before doing so, we address two existing beliefs, which we believe have disrupted thinking in this field. First, there is currently a prohibition about speaking openly about suicide, based on the notion that open discussion will encourage copycat suicide. This notion has not been proven (15) but is strongly held and inhibits detailed reporting of suicide in the press. We propose the view that informed discussion and education about suicide is likely to decrease rather than increase loss of life.

Second, it is incorrect and demeaning to characterise all individuals who experience suicidal ideation as powerless to make decisions or unable to act with consideration for others. People who complete suicide frequently research the topic and select a method based on lethality, accessibility, and personal comfort. They commonly contact police or an ambulance before acting or place warnings on doors or windows so that unprepared relatives and friends will not make the unpleasant discovery. More than a quarter leave a suicide letter describing their motives and wishes.

One public health activity has been embraced: reduction of access to lethal means (16, 17). This has included reducing access to dangerous drugs such as barbiturates, barriers at high places, the substitution of natural gas for poisonous coal gas, catalytic converters to reduce the toxicity of automobile exhaust, and the reduction in the availability of firearms. Evidence suggests these initiatives have reduced suicide, but it is not yet clear whether determined people find alternative methods to achieve their goal. Reducing access to lethal means will not be the complete answer, as alternative means are perpetually available.

We present two sets of facts. The first set would be appropriate as ‘general knowledge’—material which could be used in the education of and discussion by the general public. The second set would be appropriate for consideration by people considering suicide—this could be provided as reference material via hard copy and electronic means.

### Information for the General Community

1. Suicide has been known among all groups of people, in all places, across time. Suicide is a matter of great concern and usually great sadness. The complete prevention of suicide is probably unachievable; a realistic aim is to reduce suicide to an absolute minimum. Thus: *Suicide is a fact of life which can be minimised.*
2. People may consider suicide for many different reasons—all being associated with extreme distress. The triggers of suicide include mental disorders, but many other triggers include loss of a child, a partner, a friend, loss of a house or fortune, loss of reputation and freedom, etc. Thus: *Suicide has many different triggers.*
3. While some people who commit suicide because of mental disorder are unable to make choices, the majority of those who take their lives are able to make choices. It is disrespectful to characterise all those who have suicidal thoughts as being unable to make decisions. Most are able to decide on a method, time and place, on whether or not to leave a note, etc. Thus: *Most people who take their lives are able to make decisions.*
4. Some people believe suicide must not be openly described or discussed as this may lead to copycat suicides. This is not proven. Conversely, when the population is well informed (as with the dangers of smoking), public attitudes change and damaging behaviour may decrease. Thus: *Increased public discussion and understanding of suicide is desirable.*

### Information for the Person with Suicidal Thoughts

1. While a big part of you wants to escape or die, certainly, part of you does not want to die. The part of you that wants to die should not murder the part of you that wants to live. Thus: *Don’t murder the part of you that wants to live.*
2. Suicide may sound easy, but even with very dangerous means there is a good chance you will survive but be disabled. A gunshot to the head may blind and disfigure you, a drug overdose may cause liver damage and brain impairment, etc. Thus: *Suicide actions may leave you alive but disabled.*
3. Suicide hurts other people. It causes great harm and distress to surviving friends and relatives; sometimes they suffer grief for many years. We are all responsible for our actions (except, perhaps, some people with severe mental disorder) and need to consider the impact of our actions, not only on ourselves, but on others. Thus: *Suicide hurts other people.*
4. Many suicides happen on impulse. When suicide attempts fail to kill, individuals are commonly very pleased to have survived. Many statements have been made with the intention of encouraging people not to complete suicide impulsively. These include “Suicide is a permanent solution to a temporary problem” and “The darkest day, if you live till tomorrow, will have passed away”. Thus: *Suicidal impulses do pass, if you hold on.*
5. Many religions and philosophies state that all individuals have value. Economists tell us that we are all part of the “human capital” of our community, and we all contribute to the wealth of our community. Thus: *Suicide is a waste.*

## Conclusion

In the west, at least, there has been a widespread belief that all suicide is the result of mental disorder. This view has been resisted in some regions such as Malaysia (18). The focus on the importance of mental disorder in suicide prevention has diverted prevention activities into the medical model. These prevention activities have failed to reduce suicide rates. It is suggested that a public health approach to prevention is required. This paper offers information which would be of value to the general public: i) suicide is a fact of life which can be minimised, ii) suicide has many different triggers, iii) most people who take their lives are able to make decisions, and iv) increased public discussion/understanding of suicide is desirable, and information which may be useful to those contemplating suicide i) don’t murder the part of you that wants to live, ii) suicide actions may leave you alive but disabled, iii) suicide hurts other people, iv) suicidal impulses do pass if you hold on, and v) suicide is a waste.

It must be acknowledged that suicide is associated not only with mental disorder but also with loss of spouse, employment, liberty, and reputation (19), that suicide is frequently impulsive, and that suicide is frequently influenced by intoxication. Suicide is often unexpected, and public health measures offer a means of reducing this behaviour.
